# Promyelocytic Leukemia Protein Isoform II Promotes Transcription Factor Recruitment To Activate Interferon Beta and Interferon-Responsive Gene Expression

**DOI:** 10.1128/MCB.01478-14

**Published:** 2015-04-21

**Authors:** Yixiang Chen, Jordan Wright, Xueqiong Meng, Keith N. Leppard

**Affiliations:** School of Life Sciences, University of Warwick, Coventry, United Kingdom

## Abstract

To trigger type I interferon (IFN) responses, pattern recognition receptors activate signaling cascades that lead to transcription of IFN and IFN-stimulated genes (ISGs). The promyelocytic leukemia (PML) protein has been implicated in these responses, although its role has not been defined. Here, we show that PML isoform II (PML-II) is specifically required for efficient induction of IFN-β transcription and of numerous ISGs, acting at the point of transcriptional complex assembly on target gene promoters. PML-II associated with specific transcription factors NF-κB and STAT1, as well as the coactivator CREB-binding protein (CBP), to facilitate transcriptional complex formation. The absence of PML-II substantially reduced binding of these factors and IFN regulatory factor 3 (IRF3) to IFN-β or ISGs promoters and sharply reduced gene activation. The unique C-terminal domain of PML-II was essential for its activity, while the N-terminal RBCC motif common to all PML isoforms was dispensable. We propose a model in which PML-II contributes to the transcription of multiple genes via the association of its C-terminal domain with relevant transcription complexes, which promotes the stable assembly of these complexes at promoters/enhancers of target genes, and that in this way PML-II plays a significant role in the development of type I IFN responses.

## INTRODUCTION

Type I interferons (IFN) IFN-α/β are a large group of cytokines that play a major role in innate antiviral responses. These responses provide a crucial initial defense against invading viruses and also aid in commissioning an effective adaptive response; when a host's ability to mount an IFN response is impaired or when a virus has acquired particularly effective countermeasures against that response, severe pathogenesis typically ensues (1).

IFN-α/β expression is induced in response to various pathogen-associated molecular patterns (PAMP) (1, 2) including double-stranded RNA (dsRNA), which is produced by many viruses during their replication (3). dsRNA is recognized by pattern recognition receptors (PRRs) including RIG-I and Mda5, triggering a signaling cascade that leads ultimately to the activation of transcription factors (TFs) such as IFN regulatory factor 3 (IRF3) and NF-κB (4, 5). IRF3 is phosphorylated and moves into the nucleus (6), while NF-κB is released from its inhibitor IκB, allowing its accumulation in the nucleus (7). These TFs, together with c-jun/ATF-2, interact with the IFN-β promoter to form an enhanceosome (8). The assembled TFs recruit coactivator CREB-binding protein (CBP) or its homologue p300 (9–11), which are histone acetyltransferases (HATs) whose action promotes the assembly of the basal transcriptional machinery at the promoter.

Secreted IFN-β stimulates both the producer cells and other cells to produce IFN-α, which acts like IFN-β and so amplifies the response, as well as a large number of IFN-responsive gene (ISG) products (12). Both IFN-α and IFN-β are recognized by receptors IFNAR1 and IFNAR2 and activate the JAK-STAT signaling pathway. The phosphorylated signal transducer and activator of transcription 1 (STAT1)-STAT2 complex then associates with IRF9 to form the ISGF3 heterotrimer, which binds to the IFN-stimulated response element (ISRE) located within the promoters of most ISGs (13, 14). The ISG products establish an antiviral state in the cell (12).

The promyelocytic leukemia (PML) gene was originally identified through its involvement in a chromosomal translocation associated with acute PML (15, 16). PML protein currently has seven principal isoforms, designated PML I to VII. These isoforms contain an identical N-terminal region that includes a RING finger domain, two B-boxes, and a coiled-coil domain (RBCC, also known as the tripartite motif, TRIM) and divergent C termini as a result of differential RNA splicing (17–19).

At least some PML isoforms are essential for maintaining the integrity of nuclear substructures termed PML nuclear bodies (PML-NB), but they also play an important role in the recruitment and localization of other proteins to PML-NB. To date, more than 100 such proteins have been identified either transiently or constitutively associated with PML-NB, including CBP, p53, Sp100, Daxx, and the small ubiquitin-like modifier (SUMO) (20); strikingly, nearly one-half of these proteins are involved in transcriptional regulation.

PML proteins and PML NBs are strongly implicated in a wide variety of cell activities (21), including DNA damage and repair (22), apoptosis (23), senescence (24), and antiviral responses, including the interferon response in particular (25–27). The relationship between PML and IFN is supported first by the evidence that the PML gene itself is an ISG with ISRE and IFN-γ-activated sequence (GAS) elements in its promoter that mediate induction by type I and II IFN (28, 29). Second, ectopic expression of some isoforms of PML protein can inhibit the growth of IFN-sensitive viruses (27, 30). Third, various viruses encode proteins that disrupt PML and/or PML bodies, including the E4 Orf3 protein of human adenovirus type 5 (HAdVC-5 or Ad5) and ICP0 of herpes simplex virus 1 (HSV-1), and viruses lacking these functions are unable to overcome IFN responses (31–35). Ad5 E4 Orf3 targets PML-II specifically (36), suggesting that this isoform in particular might have a role in the IFN response, and PML-II is also one of two isoforms shown to be inhibitory to HSV-1 infection (37). Most recently, PML-IV was shown to enhance IFN-β synthesis during virus infection (25).

PML has been linked with transcription regulation in several contexts (38). A growing number of studies have demonstrated that PML participates in the regulation of cytokine signaling (39). PML protein as a whole has been implicated in type II IFN signaling, since it affected STAT-1 DNA binding (40, 41); various PML isoforms also affected IFN-γ-induced gene expression in the major histocompatibility complex class I (MHC-I) and MHC-II loci (42, 43), with PML-II binding the transcription factor CIITA (43).

We therefore sought to examine the role of PML protein in type I IFN signaling. PML-II specifically was found to play a key role in the induction of IFN-β and ISG expression. PML-II positively regulated IRF3, NF-κB, and STAT1 activities; PML-II was also found to be associated with transcriptional complexes involving these factors, and the absence of PML-II impaired the stability and DNA binding of these complexes at promoters. Finally, specific sequences in the isoform-specific C terminus of PML-II were necessary both for interaction with TFs and for ISG expression. Thus, we propose that PML-II has a role in the induction of genes of the innate immune response by mediating transcriptional complex assembly at their promoters.

## MATERIALS AND METHODS

### Cells, reagents, antibodies, plasmids, and small interfering RNA (siRNA).

Human embryonic kidney 293 (HEK293) and HeLa cells were maintained in Dulbecco's modified Eagle medium (DMEM) supplemented with 10% (vol/vol) newborn bovine serum. MRC5 cells were maintained in 10% Eagle's minimal essential medium supplemented with 10% fetal bovine serum, 2 mM l-glutamine, and 1% nonessential amino acids. Poly(I·C) was from Sigma, IFN-α was from PBL Assay Science, and Lipofectamine 2000 was from Invitrogen.

Antibodies to IRF3 (FL-425), NF-κB p65 (C-20), CBP (A-22), STAT1 p84/p91 (E-23), and p53 (DO-1) as well as goat anti-rabbit IgG–horseradish peroxidase (HRP) (sc-2054) were obtained from Santa Cruz Biotechnology; the rabbit antibody to Ser396-phospho-IRF3 was 4D4G from Cell Signaling Technology, and the antiactin antibody was from Millipore. Monospecific antipeptide sera reactive against PML-II or PML-V were previously described (44) and were provided by K.-S. Chang, M. D. Anderson Cancer Center, University of Texas. Goat anti-mouse IgG–HRP, anti-Flag–agarose beads, and mouse monoclonal F31 anti-FLAG epitope were from Sigma-Aldrich; goat anti-rabbit antibody–Alexa Fluor 594 was from Invitrogen.

The plasmids used were the following: pCI-neo-Flag-PML-II and PML-V (45, 46), a set of in-frame deletions (Δ1 to Δ3) in pCI-neo-Flag-PMLII or its ΔRBCC variant (45, 47), IFNβ-Luc (48), pISRE-Luc (Stratagene); and pcDNA3.1-HisB::lacZ (Invitrogen). RDIII/I-Luc reporter plasmid was kindly provided by Li Yong (49) and PRDII-Luc reporter by J. Mankouri (50). siRNAs targeting PML-II or PML-V (42) and a control siRNA, with no predicted targets in the human transcriptome (51), were as previously described. Alternative PML-II (sense, GGAAAGCAGAGCCCAGACUUU) or control (sense, ACGCGAAUAGCGAGCAAGCUU) siRNAs, designated “B” to distinguish them from the initially tested siRNAs, were designed for this study. Control siRNAs, at the concentrations used, did not induce significant dsRNA responses as measured by reporter assays (see below).

### Luciferase reporter assays.

Cultures were transfected with 125 pmol/ml siRNA targeting PML-II or PML-V or control siRNA for 24 h and then cotransfected with 225 ng of luciferase reporter plasmid and 25 ng pcDNA3.1-HisB::lacZ (CMV-βgal). After 24 h, cells were transfected or mock transfected with 1 μg/ml poly(I·C) for stimulation of IFN responses and harvested and lysed with passive lysis buffer (Promega) 16 h later. Lysates were assayed for luciferase and β-galactosidase activities as previously described (52). Raw data were converted to relative luciferase activity (RLA) by normalizing to the corresponding β-galactosidase activity, to correct for variation in transfection efficiency.

### Confocal immunofluorescence.

Cells grown on coverslips in 12-well culture plates were treated with siRNA and poly(I·C) as above and then processed for immunofluorescence as described previously (53) and viewed by confocal microscopy using a 63×, 1.4 numerical-aperture (NA) objective and a Leica SP2 system. All images shown are representative single images from a z stack, taken through the thickest part of the cell. Each fluorescence channel was imaged separately, and images were merged subsequently using Leica software. In some experiments, images collected in the red channel were false-colored green to improve contrast.

### IP and immunoblotting.

For immunoprecipitation (IP) and immunoblotting assays, cells cultured in 10-cm dishes were lysed in NP-40 lysis buffer (50 mM Tris-HCl [pH 8.0], 140 mM NaCl, 1% NP-40) for 10 min on ice, and extracts were sonicated and cleared by centrifugation. Immune complexes were precipitated either by specific antibody or by control IgG with rocking overnight at 4°C, followed by collection on protein A-Sepharose beads for 1 to 2 h or collected directly on anti-Flag beads by overnight incubation. Immune complexes were washed after precipitation with 10 mM Tris-HCl (pH 7.4), 150 mM NaCl, 1 mM EDTA, 1% Triton X-100, 1 mM EGTA, and 0.2 mM Na_3_VO_4_ and then released by resuspending beads in 2× SDS gel sample buffer. Proteins were separated by SDS-PAGE and analyzed by Western blotting as previously described (36).

### mRNA quantitation.

Total cell RNA was extracted by the GenElute Mammalian Total RNA Miniprep kit according the manufacturer's instructions and reverse transcribed using Superscript II (Invitrogen) and random hexamers as primer. Specific cDNAs were quantified in triplicate with an ABI Prism 7000 system or, in later experiments, an Agilent Stratagene Mx3005P system, using SYBR green quantitative PCR (qPCR) Master mix (ABI) and primer pairs for IFN-β, ISG54, and β-actin (54), ISG15 (55), ISG20 and glyceraldehyde 3-phosphate dehydrogenase (GAPDH) (56), ISG56, RANTES, and interleukin-8 (IL-8) (57), IFN-γ-inducible protein 10 (IP-10) (58), IRF7 (59), tumor necrosis factor alpha (TNF-α) (60), IL-6 (61), transferrin receptor (TFRC) (62), and succinate dehydrogenase (SDHA) (63). Quantification was based on the threshold cycle (*C_T_*) difference performed according to the ΔΔ*C_T_* method (64). Target gene expression was normalized to the mRNAs for housekeeping GAPDH or β-actin genes.

### ChIP assays.

HEK293 cells were grown to 30 to 40% confluence and then treated with siRNA and poly(I·C) as described above. Chromatin immunoprecipitation (ChIP) was carried out broadly as described previously (65). Briefly, cells were cross-linked with 1% formaldehyde for 10 min at room temperature and then harvested and resuspended in cell lysis buffer (20 mM HEPES [pH 7.9], 25% glycerol, 420 mM NaCl, 1.5 mM MgCl_2_, 0.2 mM EDTA) on ice for 20 min. Nuclei were pelleted by centrifugation in a microcentrifuge at 13,000 rpm for 10 min and lysed with nuclear lysis buffer (50 mM Tris-HCl [pH 8.0], 1 mM EDTA, 150 mM NaCl, 1% SDS, 2% Triton X-100) on ice for 10 min. DNA was sheared by sonication to a fragment size of 200 to 1,000 bp, and then lysates were diluted with IP dilution buffer (50 mM Tris-HCl [pH 8.0], 1 mM EDTA, 150 mM NaCl, 0.1% Triton X-100). After incubation with 2 μg specific antibody or control IgG at 4°C for 6 h or overnight, protein-chromatin complexes were collected on protein A-Sepharose beads, cross-linking reversed, and DNA eluted with 62.5 mM Tris-HCl [pH 6.8], 200 mM NaCl, 2% SDS, and 10 mM dithiothreitol (DTT) at 65°C for 5 h or overnight. DNA was then extracted by phenol-chloroform and analyzed by SYBR green qPCR using promoter-specific primers for IFN-β (TGCTCTGGCACAACAGGTAG and CAGGAGAGCAATTTGGAGGA; amplicon, 82 bp), ISG15 (CGCCACTTTTGCTTTTCCCT and ATAAGCCTGAGGCACACACG; 158 bp), ISG56 (TTGGGTTTCTGCAGCACTAGA and ACCTAGGGAAACCGAAAGGG; 150 bp), protein kinase R (PKR) (TACCCCAATCCCGTAGCAGA and CGTTTTCCCCTTGGACTCCG; 82 bp), and p21 (ATCCCTATGCTGCCTGCTTC and TCTCCTACCATCCCCTTCCT; 184 bp). An amplicon located 10.3 kbp from the ISG56 promoter ISRE was also tested (CTCTGCCTATCGCCTGGATG and CCTGCCTTAGGGGAAGCAAA; 77 bp). All primers were designed with the NCBI primer designing tool and were validated for amplification efficiency and for specificity using standard curve and melt curve analyses, respectively.

## RESULTS

### Depletion of PML-II reduces IFN-β expression.

**FIG 1:**
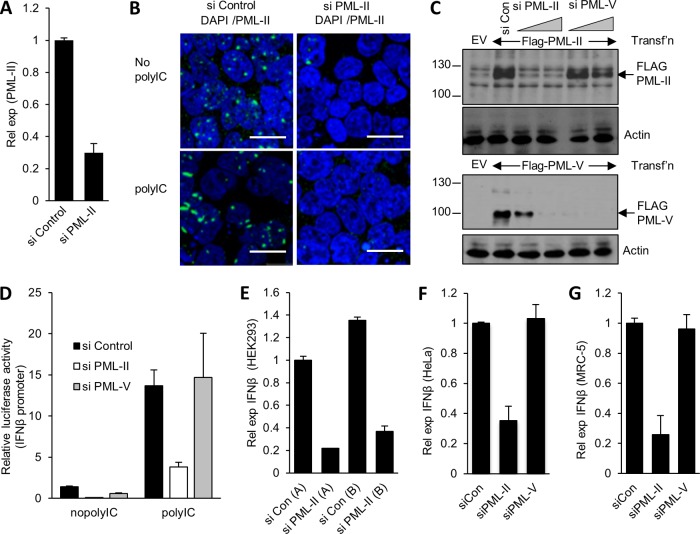
Depletion of PML-II reduces IFN-β expression. (A) HEK293 cells were transfected with 125 pmol/ml siRNA as indicated and, after 48 h, transfected with 1 μg/ml poly(I·C) for 16 h. PML-II mRNA levels in total RNA were determined by RT-qPCR and normalized to β-actin mRNA levels and are shown here relative to values obtained from control siRNA-treated cells. (B) HEK293 cells grown on coverslips were treated with siRNA and poly(I·C) as described for panel A and then fixed and stained with PML-II antibody. Images shown are overlays of DAPI (4′,6-diamidino-2-phenylindole) (blue) and PML-II staining (green) and are representative of multiple fields that were randomly selected based on DAPI fluorescence. Scale bar, 20 μm. (C) HEK293 cells were cotransfected with 450 ng pCI-neo and either 50 ng Flag-PML-II (upper panels) or Flag-PML-V (lower panels), alongside 75 pmol/ml or 125 pmol/ml of the siRNAs indicated. Control siRNA cells were treated with 125 pmol/ml siRNA. Forty-eight hours later, cells were harvested and analyzed by SDS-PAGE and Western blotting with anti-FLAG and antiactin antibodies. Protein sizes are indicated in kDa. Empty vector (EV) lane, background detection by the anti-FLAG antibody. (D) HEK293 cells were treated with the siRNAs indicated for 24 h, cotransfected with IFN-β-Luc and CMV-βgal plasmids for a further 24 h, and then transfected with 1 μg/ml poly(I·C) for 16 h before being harvested for reporter assays. Error bars indicate the standard deviations (SD) from the means for at least three biological replicates within an experiment. (E) HEK293 cells were treated with the siRNAs indicated for 48 h and then stimulated with poly(I·C) as described for panel A. IFN-β mRNA levels in total RNA were determined by RT-qPCR and normalized to β-actin mRNA levels and are shown here relative to values obtained from control siRNA-treated, poly(I·C)-induced cells. In this experiment, poly(I·C) treatment achieved an 8,000-fold stimulation of IFN-β mRNA levels. Data are the means ± SD of three replicate values from one representative of three experiments. (F) HeLa cells were treated and assayed for IFN-β mRNA levels, and data were analyzed and presented as described for panel E. Poly(I·C) treatment achieved a 1,000-fold stimulation of IFN-β mRNA levels. (G) MRC5 cells were treated with 50 pmol/ml siRNA for 72 h prior to transfection with 1 μg/ml poly(I·C). Twenty-four hours later, total RNA was analyzed for IFN-β expression by RT-qPCR and data were analyzed as described for panel E. Data are the means ± SD from five replicates in two independent experiments. A fold stimulation value for the effect of poly(I·C) could not be obtained, as basal IFN-β expression was undetectable.

In order to investigate the function of PML-II in interferon expression, we first established conditions for transient knockdown of PML-II using previously described small interfering RNA (42). Endogenous PML-II mRNA was significant reduced, to about 20 to 30% of control levels, by siRNA treatment (Fig. 1A). At the protein level, this knockdown protocol reduced the prominent foci of PML-II nuclear fluorescence to undetectable levels in >90% of cells (Fig. 1B). To confirm the isoform specificity of the knockdown, we overexpressed Flag-tagged PML-II or PML-V and observed the reduction in each protein following specific siRNA treatment. Substantial reductions were seen in amounts of the major 106-kDa exogenous PML-II protein form in siPML-II-transfected HEK293 cells compared with either control siRNA or siPML-V treatment (Fig. 1C, top); conversely, PML-V was efficiently removed by siPML-V while being relatively more resistant to siPML-II (Fig. 1C, bottom). The bands in the empty vector (EV) lane are background detected by the anti-FLAG antibody.

The effect of reducing PML-II levels on IFN-β expression was first tested using a luciferase reporter assay. As expected, upon stimulation with poly(I·C), the activity of the IFN-β promoter was significantly increased, but prior depletion of PML-II resulted in a significant decrease in this level of induced promoter activity, to levels approximately 25% that of the control (Fig. 1D). In contrast to PML-II, the selective removal of PML-V by siRNA (Fig. 1C) had no effect on IFN-β promoter activity (Fig. 1D). To confirm that the effects of PML-II siRNA in reporter assays reflected the behavior of the endogenous IFN-β promoter, endogenous IFN-β mRNA levels were tested by reverse transcriptase (RT) qPCR. Amounts of mRNA induced by poly(I·C) in HEK293 cells (Fig. 1E) and in HeLa cells (Fig. 1F) were significantly reduced by depleting PML-II. Involvement of off-target effects in this reduction was excluded by demonstrating that a second siRNA targeting an unrelated sequence in the PML-II mRNA [siPML-II (B)] had the same effect on IFN-β mRNA levels as the initially tested siPML-II RNA (A) (Fig. 1E). To extend these findings to normal cells, a strain of human lung fibroblast cells (MRC5 cells) was also tested. As in immortalized cells, depleting PML-II significantly reduced the induced IFN-β mRNA level (Fig. 1G). This effect was specific to removal of PML-II, as depletion of PML-V, which is expressed from a distinct but closely related mRNA, had no effect on IFN-β expression in either HeLa or MRC-5 cells (Fig. 1F and G). Taken together, these data indicate that expression of the IFN-β gene upon poly(I·C) stimulation is significantly dependent on the presence of PML-II.

### Depletion of PML-II impairs IRF3 and NF-κB activities.

**FIG 2:**
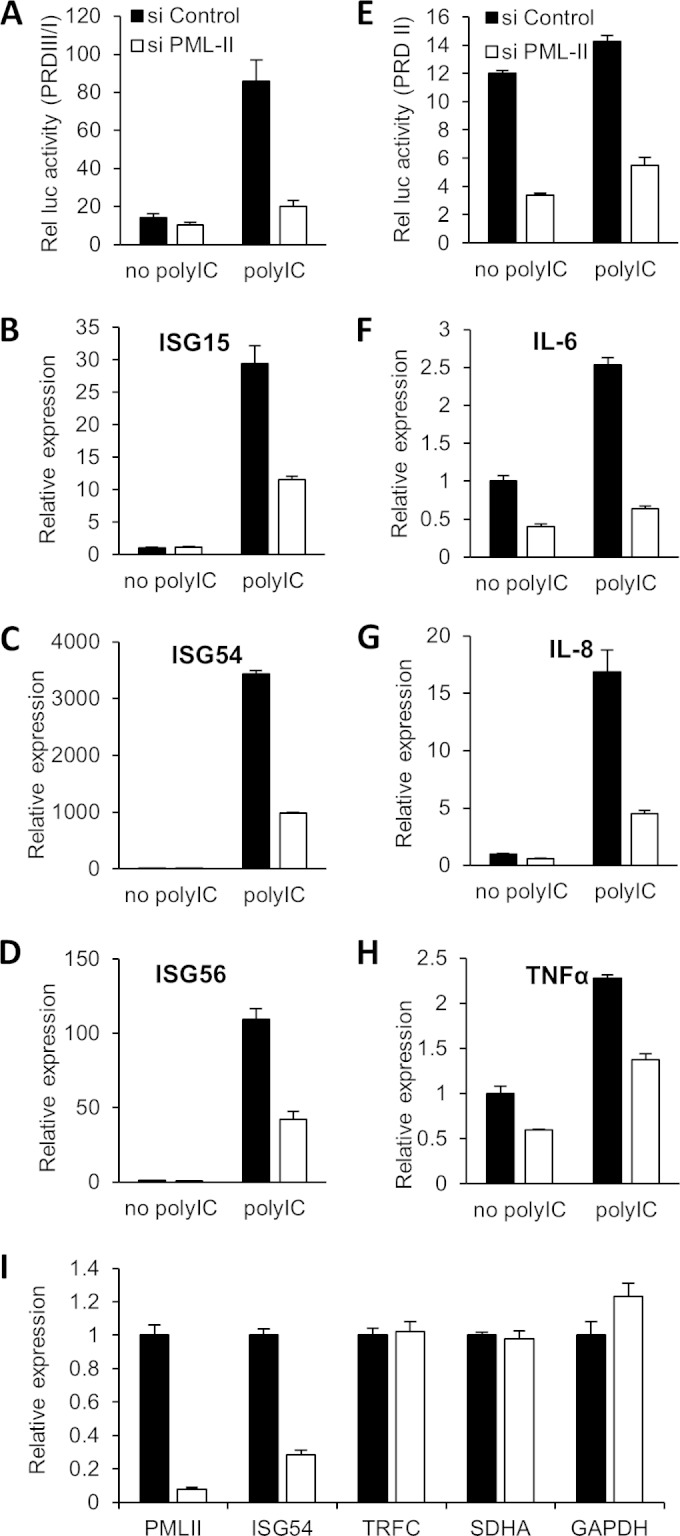
Depletion of PML-II impairs activities of IRF3 and NF-κB. (A, E) HEK293 cells were treated with siRNA as indicated for 24 h, cotransfected with CMV-βgal and PRDIII/I-Luc (A) or PRDII-Luc (E) for 24 h, stimulated with 1 μg/ml poly(I·C) or control treated for 16 h, and then assayed for reporter activity. Error bars indicate the standard deviations from the means for at least three replicates. (B to D, F to H) HEK293 cells were treated with siRNA for 48 h and stimulated with poly(I·C) as described for panel A. Total RNA was analyzed for specific mRNA levels by RT-qPCR. mRNA levels are displayed relative to those observed in control siRNA-treated cells without stimulation. Error bars indicate the standard deviations of the means from at least three replicates within an experiment. (I) As for panels B to D and F to H, except values were normalized in each case to the level observed in control siRNA-, poly(I·C)-treated cells; there was no significant difference in expression levels with or without stimulation.

The stimulation of IFN-β expression requires the coordinated activation of multiple transcription factors. Among these, IRF3 and NF-κB are critically important, acting through promoter subelements PRDIII/I and PRDII, respectively (5, 8, 66, 67). Given the effect of PML-II depletion on IFN-β expression, we tested the activities of IRF3 and NF-κB individually under PML depletion conditions using PRD-III/I- and PRDII-driven luciferase reporters. As expected, the activity of the IRF3-responsive reporter, PRDIII/I, was greatly increased upon stimulation with poly(I·C); remarkably, prior depletion of PML-II almost completely abolished this increase (Fig. 2A). To confirm the effect of PML-II depletion on IRF3 activity, we also measured the induction of mRNA from a series of endogenous IRF3-responsive genes, including ISG15, ISG54, and ISG56 (11, 68), with or without PML-II depletion (Fig. 2B to D); mRNA expression from these genes was significantly dependent on PML-II.

Unlike the IRF3-responsive construct, the NF-κB-responsive reporter had significant basal activity in HEK293 cells, and this activity was only marginally increased by poly(I·C) stimulation. However, removal of PML-II still reduced its activity substantially, with or without stimulation (Fig. 2E). Similarly, the depletion of PML-II reduced poly(I·C)-induced IL-6, TNF-α, and IL-8 mRNA levels (Fig. 2F to H), which are established NF-κB-dependent genes (60, 69). Collectively, these results indicate that depletion of PML-II impairs promoter activation by both IRF3 and NF-κB. To exclude the possibility that PML-II was generally required for efficient gene expression, we also tested the expression of a number of control genes. Under conditions of PML-II depletion and poly(I·C) stimulation, expression of a representative ISG was greatly reduced as expected, while expression of three housekeeping genes was unaffected (Fig. 2I).

### PML-II plays a role in the JAK/STAT signaling pathway.

**FIG 3:**
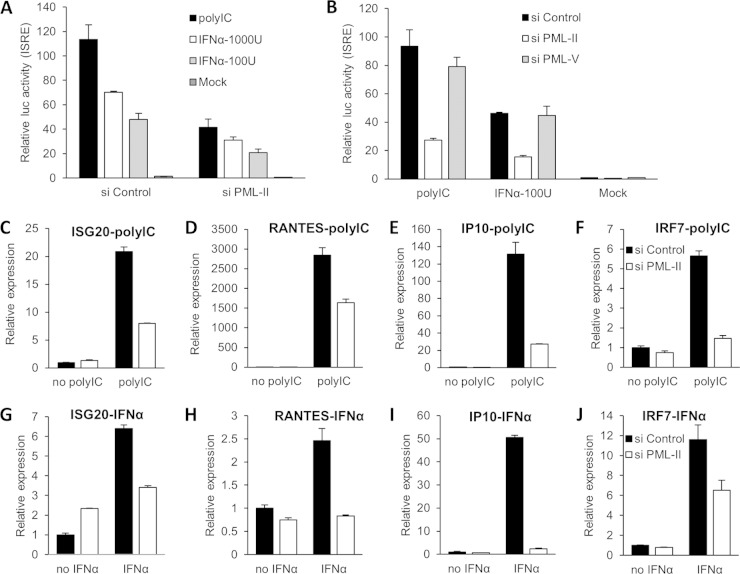
PML-II depletion reduces expression of ISGs. (A, B) HEK293 cells were transfected with siRNA as indicated for 24 h and then cotransfected with ISRE-Luc and CMV-βgal plasmids for 24 h. Following either poly(I·C) transfection for 16 h or IFN-α treatment for 8 h, cell lysates were assayed for reporter activity. Data shown are the means ± SD from at least three replicates. (C to J) HEK293 cells were treated with siRNA for 48 h and stimulated with either poly(I·C) for 16 h (C to F) or 1,000 units/ml IFN-α for 8 h (G to J). Total RNA was analyzed for specific mRNA levels by RT-qPCR. mRNA levels are displayed relative to those observed in control siRNA-treated cells without stimulation. Quantification was performed as described for Fig. 1E. Data shown are the means ± SD from at least three replicates within an experiment.

Impaired IFN-β expression due to knockdown of PML-II should affect downstream signaling and thus the activation of IFN-stimulated genes. Therefore, it was expected that activation of an ISRE-dependent promoter by poly(I·C) should be indirectly inhibited by PML-II depletion, as was observed (Fig. 3A). More surprisingly, when IFN-α, which directly activates the JAK-STAT signaling pathway, was used as the inducer, activation of the ISRE was again strongly inhibited by PML-II depletion (Fig. 3A). This effect was specific to depletion of PML-II since depleting PML-V had no effect on ISRE activity when stimulated by either inducer (Fig. 3B). Thus, a significant part of the effect of PML-II depletion during poly(I·C) induction is attributable to a direct effect on the JAK-STAT pathway.

To further investigate the importance of PML-II to IFN-β downstream signaling, induced mRNA levels from a series of ISGs including ISG20, RANTES (CCL5), IP-10 (CXCL10), and IRF7 were measured. Expression induced by either poly(I·C) (Fig. 3C to F) or IFN-α (Fig. 3G to M) was in all cases significantly reduced by depleting PML-II. Thus, PML-II depletion reduces the expression of numerous ISGs, both via its effect on IFN-β expression and due to a direct impact on signaling via the JAK/STAT pathway.

### PML-II has little effect on cytoplasmic signaling events leading to IFN-β induction.

**FIG 4:**
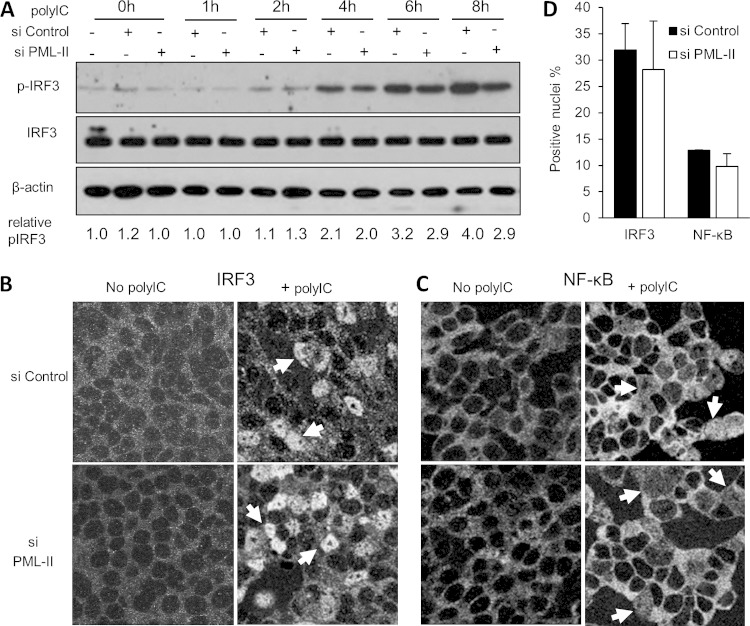
PML-II has limited effects on cytoplasmic events in IFN-β induction. (A) HEK293 cells were transfected with 125 pmol/ml PML-II or control siRNA for 48 h and then stimulated by transfection with 1 μg/ml poly(I·C) (*t* = 0) before collection at the indicated time points. Samples were separated by SDS-PAGE, blotted, and probed with antibody to phosphorylated IRF3, total IRF3, or β-actin. Band intensities in digitized images were quantified using QuantityOne software (Bio-Rad); the amounts of pIRF3 detected are shown relative to total IRF3. (B) HEK293 cells grown on coverslips were treated with siRNA as described for panel A and then transfected with 1 μg/ml poly(I·C) or mock transfected for a further 16 h before fixation and detection of IRF3 by immunofluorescence. Arrows indicate examples of nuclei that showed positive staining for IRF3 following poly(I·C) stimulation. (C) As for panel B but detecting NF-κB. (D) Quantification of IRF3 and NF-κB nuclear staining. All nuclei in a field were detected by DAPI staining, and the proportion showing positive staining for IRF3 or NF-κB was determined manually over 2 or 3 fields of view.

In principle, the role of PML-II in the induction of IFN-β expression could be at any point from PAMP sensing through the resulting signaling cascade to the assembly of the enhanceosome at the IFN-β promoter. To investigate where PML-II acted in this pathway, we first examined IRF3 phosphorylation and IRF3 and NF-κB nuclear translocation, which have been described as key steps in signal transduction following PAMP recognition (5, 6, 70). Neither poly(I·C) stimulation nor siRNA transfection affected the expression of total IRF3 (Fig. 4A). The amount of phosphorylated (activated) IRF3 increased gradually with the duration of poly(I·C) stimulation; depletion of PML-II did reduce the accumulation of phospho-IRF3 somewhat (Fig. 4A), although this effect was modest and was apparent only with longer periods of stimulation, suggesting that it was a secondary consequence of the changes in ISG expression observed. Some phosphorylated IRF3 was also detected in unstimulated cells. This may reflect a requirement to maintain a basal level of ISG expression in order to give a rapid antiviral response in the initial stages of infection, though NF-κB has been shown to be more important than IRF3 for this (71, 72).

Stimulation with poly(I·C) also caused the expected movement of IRF3 into the nucleus; the number of IRF3-positive nuclei increased from 0% to between 30% and 40% by 16 h; consistent with the limited effect on pIRF3 level, depletion of PML-II had no significant effect on the fraction of cells showing IRF3 nuclear localization following poly(I·C) treatment (Fig. 4B and D). For NF-κB, the effect of poly(I·C) stimulation on localization was less striking. Only 10% of nuclei stained positive for NF-κB (p65) after 16 h, and in these cells, there was still predominantly cytoplasmic fluorescence (Fig. 4C and D). As with IRF3, there was no significant difference in the extent of NF-κB nuclear translocation in the presence or absence of PML-II (Fig. 4C and D). This is consistent with the lower activity of NF-κB relative to IRF3 in the IFN-β subdomain reporter assays in this cell type (Fig. 2A and E). Collectively, these results show that PML-II has only limited effects on the cytoplasmic signaling events leading to IFN-β induction; the more profound effects of PML-II depletion on gene expression must therefore be accounted for by events in the nucleus.

### PML-II interacts with transcriptional complexes.

**FIG 5:**
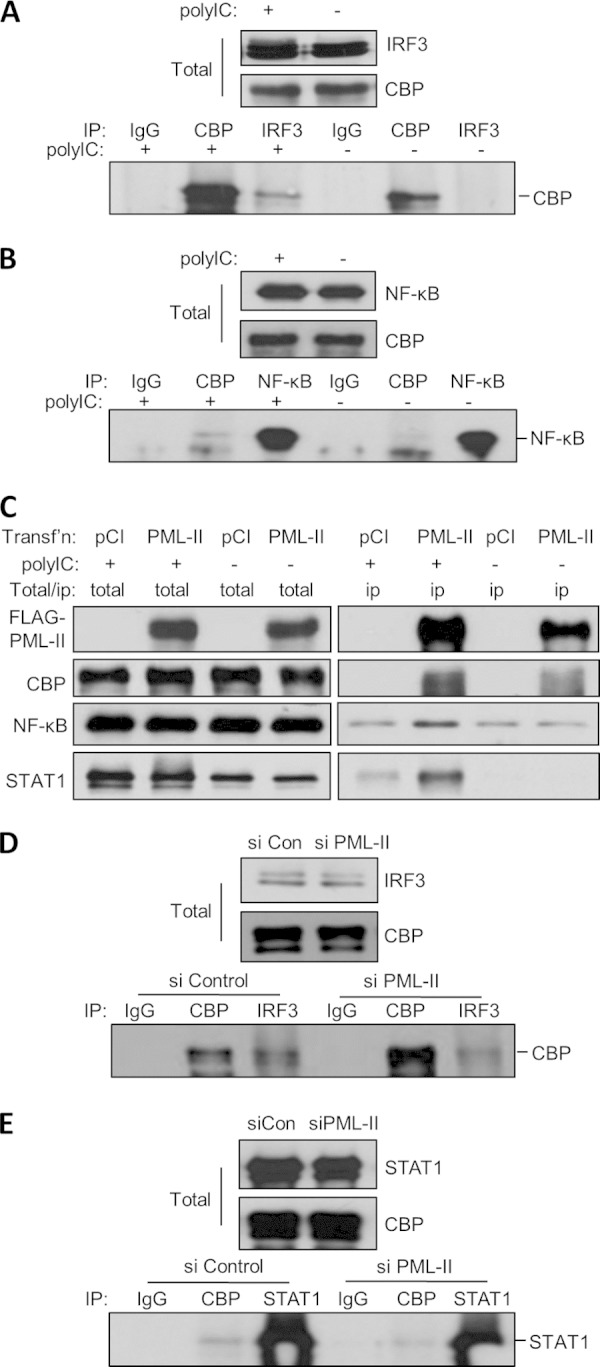
PML-II interacts with transcriptional complexes. (A, B) HEK293 cells were mock stimulated or transfected with 1 μg/ml poly(I·C) for 16 h and then lysed with 0.5% NP-40 lysis buffer. A portion of each cell lysate was retained for direct analysis by Western blotting, while equal parts of the remainder were incubated with IRF3, NF-κB (p65), or CBP antibodies or with control IgG and precipitated with protein A-Sepharose beads; proteins in total or immunoprecipitated samples were separated by SDS-PAGE, and IRF3, NF-κB, or CBP was detected by Western blotting. (C) HEK293 cells were transfected with 250 ng/ml Flag-PML-II plasmid or pCI-neo empty vector for 24 h and then stimulated with poly(I·C); lysates were prepared and immunoprecipitated with anti-Flag beads, and precipitates and total lysates were analyzed for Flag-PML, CBP and NF-κB, and STAT1 as described for panel A. (D, E) HEK293 cells were treated with siRNA and then stimulated with poly(I·C) as described for Fig. 1A. Lysates were prepared and immunoprecipitated with IRF3 or CBP antibodies, and precipitates and total lysates were analyzed for IRF3, STAT1, and CBP as described for panel A.

Interaction with different partner proteins such as specific transcription factors is important for PML involvement in various cellular activities (38, 73–76). The assembly of transcription factors (TFs), including IRF3 and NF-κB, and coactivators CBP/p300 into a transcription complex at the IFN-β promoter is a key step for IFN-β induction (5, 6, 10, 11). It is also known that CBP can be recruited to PML-NB and that it interacts with PML (73, 77), suggesting that PML might also associate with the transcriptional complex involved in IFN-β activation. We therefore examined the association of PML-II with IRF3, NF-κB, and CBP. As expected from earlier reports, poly(I·C) induced the association of IRF3 and NF-κB with CBP (Fig. 5A and B) (11, 78). Overexpressed Flag-tagged PML-II also associated with CBP in both stimulated and unstimulated cells, consistent with previous observations (Fig. 5C) (73, 77). Importantly, in these Flag-PML-overexpressing cells, poly(I·C) stimulation induced a greater association of NF-κB with complexes precipitated by Flag beads than what was seen with no stimulation or in empty vector-transfected cells, although some nonspecific background precipitation was observed (Fig. 5C). Similarly, poly(I·C) stimulation induced a strong interaction of Flag-PML-II with complexes that contained STAT1, a component of the transcription factor ISGF3, whereas in unstimulated cells there was no detectable interaction (Fig. 5C). In light of these results, we next tested whether depletion of PML-II affected the stability of the induced association between these transcriptional complex components. PML-II depletion substantially reduced the association between CBP and IRF3 (Fig. 5D, compare lanes labeled IRF3) or STAT1 (Fig. 5E, compare lanes labeled CBP); quantitation of these effects showed that the CBP-IRF3 interaction was reduced to 66% and the CBP-STAT1 interaction to 62% of their respective controls. Taken together, these data indicate the formation of transcriptional complexes during poly(I·C) stimulation that involve PML-II, CBP, and three DNA-binding TFs, IRF3, NF-κB, and STAT1. They further show that PML-II contributes to the stable association of TFs with CBP.

### PML-II alters transcription factor binding at promoters.

**FIG 6:**
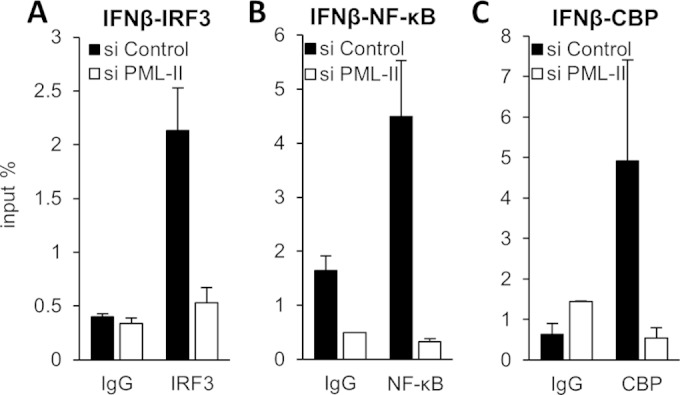
Knockdown of PML-II impairs IRF3, NF-κB, and CBP recruitment to the IFN-β promoter. HEK293 cells were transfected with PML-II or control siRNA for 48 h and stimulated with poly(I·C) for 16 h for IRF3, 10 h for NF-κB, or 10 h for CBP. Cells were then fixed and subjected to ChIP with IRF3 (A), NF-κB (B), or CBP (C) antibodies, in each case in parallel with a normal IgG control precipitation on an equal volume of extract. Precipitated DNA was assayed for IFN-β promoter sequences by SYBR green qPCR. Precipitated DNA is expressed as the mean percentage of the amount of that DNA present in the extract volume subjected to precipitation ± SD from one representative of at least two experiments.

TF binding at promoters or enhancers is essential for gene transcription. We next examined the effect of PML-II depletion on the binding of TFs at the IFN-β promoter by chromatin immunoprecipitation (ChIP). In line with the observed effects on promoter activity, depletion of PML-II led to significant reductions in IRF3 and NF-κB binding at the IFN-β promoter (Fig. 6A and B). Previous studies have demonstrated that the TFs assembled at the IFN-β promoter can recruit the coactivators CBP/p300 and that this is important for activation (5, 6, 10, 11). Therefore, the absence of this PML isoform might also affect CBP binding to the IFN-β promoter. Indeed, there was a significant loss of CBP binding at the promoter in the absence of PML-II (Fig. 6C). Thus, the knockdown of PML-II affects TF and CBP recruitment to the IFN-β promoter, which accounts for the severely reduced expression of the gene when induced under conditions of PML-II depletion.

**FIG 7:**
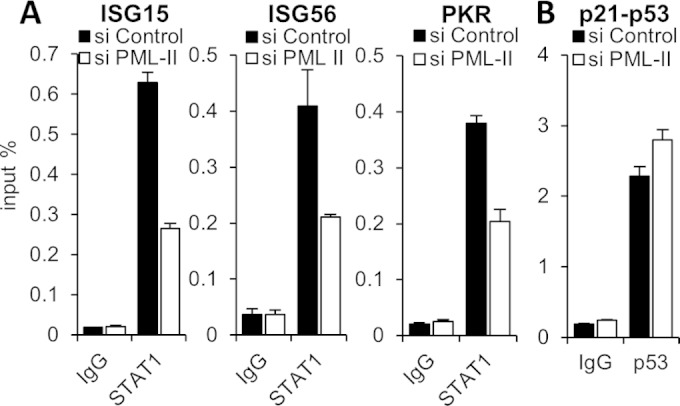
Removal of PML-II affects transcription factor binding at the ISRE elements of ISGs. (A) HEK293 cells were transfected with PML-II or control siRNA for 48 h and then transfected with poly(I·C) for 16 h. Cells were fixed and subjected to ChIP with STAT1 antibodies in parallel with a normal IgG control precipitation on an equal volume of extract. Precipitated DNA was assayed for ISG15, ISG56, and PKR promoter sequences by SYBR green qPCR, and data were processed as for Fig. 6. (B) HEK293 cells treated with siRNA as described for panel A were fixed and subjected to ChIP analysis using p53 antibodies and qPCR primers specific for the p21 promoter.

The expression of ISGs requires ISGF3 assembly and binding to ISRE elements. We therefore considered that PML-II might also affect the binding of ISGF3 to the ISRE, which was tested by ChIP analysis for its STAT1 component on three representative ISG promoters. Consistent with the effect of PML-II depletion on ISG expression, STAT1 binding to these promoters was decreased in the absence of PML-II (Fig. 7A). Finally, to test whether PML-II more generally affected TF binding to chromatin, we examined the binding of p53 to a well-characterized target, the p21 promoter; p53 is known to be bound to this promoter even in the uninduced state (79). PML-II knockdown had no effect on this interaction (Fig. 7B). Thus, taken together, our results indicate that PML-II specifically and positively regulates TF binding at ISRE elements and at the IFN-β promoter, correlating with its effects on expression of these genes.

### The PML-II unique C-terminal domain is required for TF binding and enhanced gene expression.

**FIG 8:**
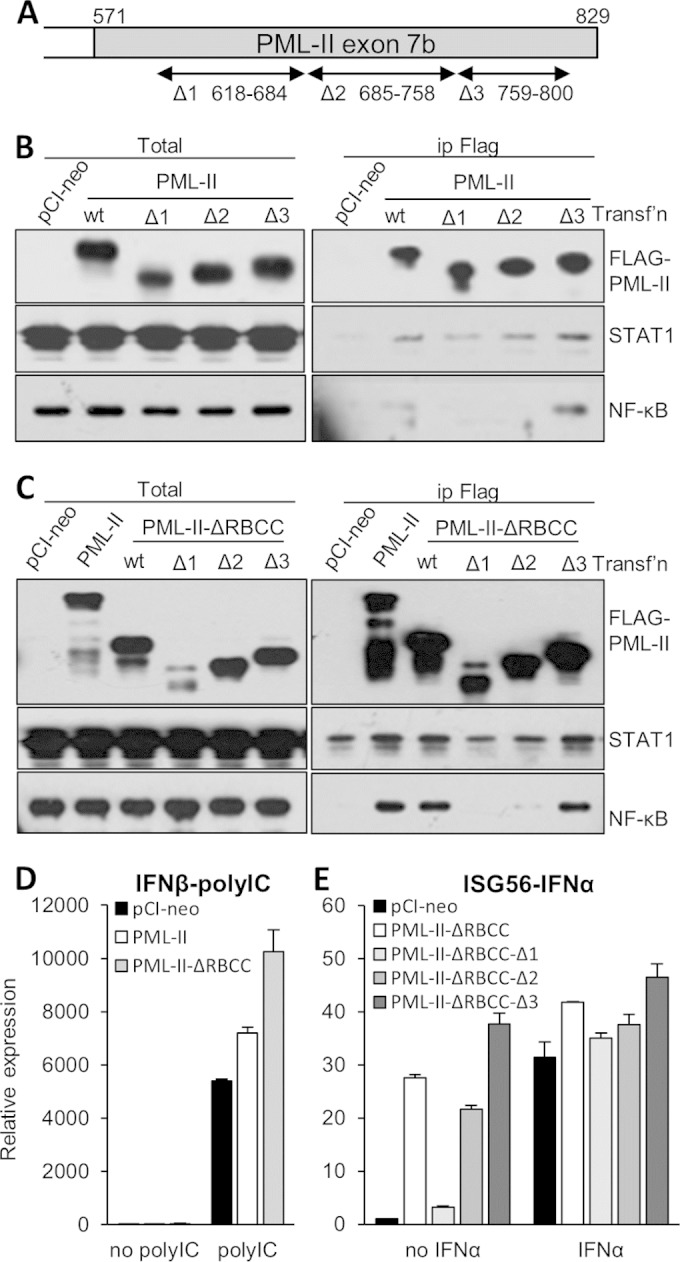
PML-II functions in gene expression via its unique C-terminal domain. (A) Representation of the C-terminal region of PML-II showing the positions of deletion mutations (47). (B) HEK293 cells were transfected with 250 ng/ml Flag-PML-II wild-type or deletion mutant plasmid or pCI-neo empty vector as indicated for 48 h and then stimulated with poly(I·C), and lysates were prepared and immunoprecipitated with anti-Flag beads. Precipitates and total lysates were analyzed for Flag-PML, NF-κB (p65), and STAT1 by Western blotting. (C) As described for panel B but using ΔRBCC variants of each PML-II plasmid. (D, E) HEK293 cells were transfected with plasmids as described for panels B and C. Following 1 μg/ml poly(I·C) (B) or 1,000 units/ml IFN-α stimulation (C) for 16 h, total RNA was analyzed for specific mRNA levels by RT-qPCR. mRNA levels are displayed relative to those observed in control siRNA-treated cells without stimulation. Quantification was performed as described for Fig. 1. Data shown are the means ± SD for at least three replicates within an experiment.

PML isoforms have distinct functions due to their different C-terminal domains, which are important for their interaction with partner proteins (80). For example, previous work in our laboratory showed that a 40-amino-acid residue segment of the PML-II C-terminal domain conferred an interaction between this protein and Ad5 E4 Orf3 (47). The role of PML-II unique C-terminal sequences in binding cellular TFs was tested by coimmunoprecipitation using a set of Flag-tagged PML-II deletion mutants, PML-II-Δ1, PML-II-Δ2, and PML-II-Δ3 (47), recloned into a full-length PML-II background (Fig. 8A). Full-length PML-II associated with NF-κB and STAT1 (Fig. 8B), as expected from the results shown in Fig. 5C. Deletion Δ3 was at least as effective as the full-length protein in these interactions; however, both Δ1 and Δ2 mutants essentially lost the association with NF-κB, while Δ1 had somewhat reduced STAT1 binding (Fig. 8B).

The N-terminal RBCC domain of PML protein mediates multimerization with other PML isoforms (reviewed in reference 19). To exclude the possibility that PML-II binding with other isoforms was contributing to the observed interactions with NF-κB and STAT1, TF binding to Δ1 to Δ3 mutants was reanalyzed using constructs with the PML RBCC domain deleted. As expected for a function unique to PML-II, deletion of the N-terminal RBCC domain did not affect binding to NF-κB or STAT1 (Fig. 8C). Deletion Δ1 abolished and Δ2 significantly diminished specific binding to NF-κB, while the deletion Δ3 mutant again retained full interaction capability (Fig. 8C) and the Δ1 mutant also showed a significantly reduced association with STAT1. These results match closely those seen for full-length PML-II.

PML-II overexpression only modestly potentiates activation of endogenous IFN-β expression by poly(I·C), but this effect is increased when the RBCC domain is deleted (Fig. 8D). Consistent with the TF binding data (Fig. 8B and C), PML-II Δ1 in a ΔRBCC background had impaired ability to stimulate (or to hyperstimulate in the presence of IFN-α) the expression of a representative ISG, ISG56 (Fig. 8E). Collectively, these results indicate that the PML-II unique C-terminal domain is essential for its binding with TFs and for activation of gene expression in the IFN response.

### PML-II associates with target promoters.

**FIG 9:**
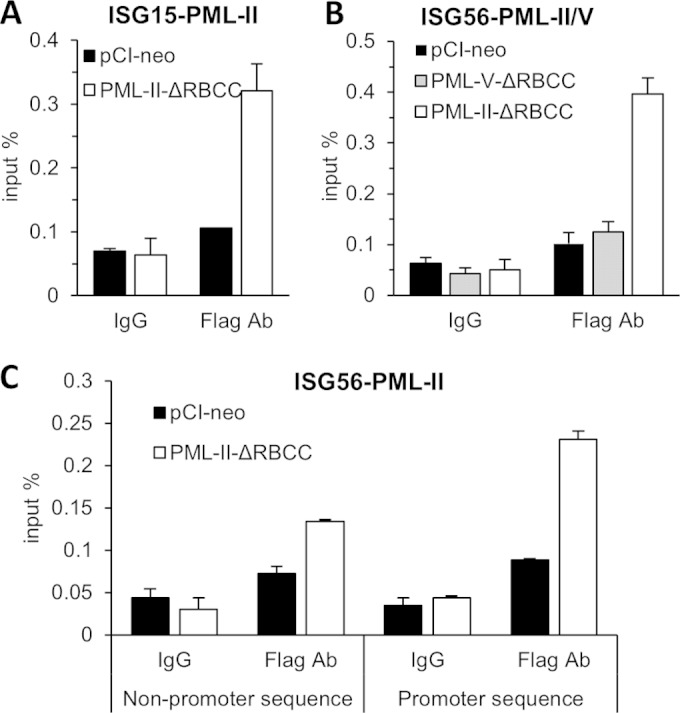
PML-II associates with target promoters. HEK293 cells were transfected with 250 ng/ml Flag-PML-II-ΔRBCC, Flag-PML-V-ΔRBCC, or pCI-neo plasmids, and 48 h later, cells were transfected with poly(I·C) for 16 h. Cell lysates were subjected to ChIP with normal IgG or anti-Flag antibody. Precipitated DNA was assayed by SYBR green qPCR for ISG15 and ISG56 promoter or nonpromoter sequences. Results are presented as means ± SD from triplicate determinations within an experiment.

PML-II interacts with specific TFs and CBP, and this complex is induced by poly(I·C) stimulation (Fig. 6). Having shown that the ΔRBCC form of PML-II could both bind specific TFs and increase the activity of target promoters (Fig. 8), we finally sought to test whether PML-II ΔRBCC was recruited to ISG promoters by ChIP analysis. It was necessary to use exogenously expressed PML for this experiment to provide a Flag tag for immunoprecipitation, and we elected to use the ΔRBCC form to avoid effects of potential interactions between exogenous and endogenous PML via their RBCC domains. These experiments thus involve significant overexpression of the FLAG-tagged protein relative to endogenous levels of PML-II, though the ratio in individual cells will vary (45). As predicted from our protein coprecipitation studies, results showed that in poly(I·C)-stimulated cells there was significant association of Flag-PML-II ΔRBCC with the ISG15 and ISG56 promoters (Fig. 9A and B). This interaction was specific to PML-II since an equivalent Flag-tagged PML-V protein, the knockdown of which had no effect on IFN-β or ISG expression (Fig. 1D and 3B), showed no association with the ISG56 promoter (Fig. 9B). The interaction with PML-II was focused on the promoter region, since amplification of a target taken from elsewhere in the ISG56 gene gave a significantly lower signal (Fig. 9C). Thus, the unique C-terminal region of PML-II associates specifically with target promoters.

## DISCUSSION

PML protein has been demonstrated to be involved in a large number of cellular processes, including antiviral defense and transcriptional regulation (21, 26, 81). However, although a role for PML in the type I IFN response has been implied by many previous studies, a mechanism has remained elusive. In this study, we investigated the function of PML in controlling the type I interferon response. Our data show that one isoform, PML-II, acts as a positive regulator of both IFN-β transcription and transcription of downstream response genes that are activated by type I IFN. This positive effect is achieved via PML-II interacting with multiple transcriptional complexes and increasing their binding at target promoters.

Our study used siRNAs to specifically target particular PML isoforms. PML-II is expressed from an mRNA that has been spliced to remove an intron that, when retained as part of the mRNA, encodes the unique C terminus of PML-V. Thus, in a PML-V mRNA the sequence encoding the C terminus of PML-II remains unused in the 3′ untranslated region. Therefore, any siRNA targeted toward the PML-II unique 3′ exon necessarily also targets PML-V mRNA. However, our results show that while depletion of PML-II significantly reduced expression of IFN-β and ISGs, depletion specifically of PML-V had no effect (Fig. 1D, F, and G and 3B). Thus, the biological effect of PML-II siRNA is not due to effects on PML-V expression. The PML gene also encodes PML-III from an mRNA that is very similar to PML-II mRNA, differing from it only by the retention of an additional 40 nucleotides (nt) of sequence at the 5′ end of its unique 3′ exon. We have not specifically depleted PML-III alone and therefore cannot formally exclude that it plays a role in the expression of IFN-β and ISGs. However, the PML-III unique C terminus is short and poorly conserved in comparison with PML-II (71 versus 259 residues) (47), it is expressed at very low levels, particularly in normal cells (82), in which we still observed a strong negative effect of PML-II siRNA treatment on gene expression, and its overexpression has been reported to have no effect on IFN-α-activated gene expression (41), so we consider it unlikely that PML-III is significant in this context.

We found that PML-II was necessary for efficient IFN-β transcription in response to stimulus with poly(I·C), a form of dsRNA that is recognized by cells as a PAMP, an effect mediated through increased TF binding at the IFN-β promoter. Recently, exogenous PML-IV (but not PML-II) was reported to potentiate IFN-β expression in response to infection with vesicular stomatitis virus by sequestering at PML-NBs the peptidyl-prolyl *cis/trans*-isomerase (Pin1) that is needed for the degradation of the key activating TF, phosphoIRF3 (25). This study found, as we do, that exogenous PML-II overexpression has little positive effect on the production of IFN-β mRNA in response to stimulus; however, the study did not test whether selective removal of PML-II would abrogate the potentiating effect of PML-IV overexpression, which our study would predict. Thus, the two studies reveal different PML functions in the IFN response that are associated with distinct PML isoforms.

Our data show that PML-II promotes the binding of STAT1 to ISG promoters, which occurs as part of the ISGF3 complex with STAT2 and IRF9. A role for PML protein in the regulation of transactivation by STAT1 homodimer in response to type II IFN-γ-mediated signaling was reported previously; however, contradictory results as to an activating or inhibitory role for PML were obtained by three different groups (40, 41, 43). Choi et al. found that activity of a GAS reporter plasmid induced by IFN-γ was increased in PML-null mouse embryonic fibroblasts (MEFs), as was the amount of phospho-STAT1 and GAS-binding activity of STAT1 homodimer from nuclear extracts *in vitro*, while, using the same cell system as well as siRNA knockdown in human cells, El Bougrini et al. found the exact opposite, and they further found that overexpression of any nuclear PML isoform potentiated the response to IFN-γ. In agreement with the latter work, Ulbricht et al. found that PML depletion reduced the transcriptional upregulation of MHC-II expression in response to IFN-γ in primary human fibroblasts or Hep2 cells. Our present study does not address or resolve this conflict; however, we did find that PML-II positively regulated the JAK/STAT signaling pathway in response to both poly(I·C) and IFN-α stimulation. El Bougrini et al. also tested the response of an ISRE reporter to IFN-α in 293 cells following knockdown of PML; in contrast to our results, they found only a modest reduction in response. It is likely that this difference relates to their use of a total PML knockdown strategy, whereas we targeted PML-II specifically; the absence of multiple PML isoforms is likely to affect the cells in a complex way, with the net result being the sum of multiple effects.

We also found that the activity of NF-κB was impaired by depleting PML-II in both stimulated and unstimulated cells. The latter result supports and extends a recent microarray analysis that showed that the knockdown of all PML isoforms suppressed the expression of a group of NF-κB-dependent genes such as IL-6 and IFN-γ-inducible protein 10 (IP-10) genes (83). The weak activation of NF-κB compared with that of IRF3 in response to poly(I·C) stimulation in HEK293 cells may reflect the inhibitory effect on its activation of the Ad5 E1B 19K protein present endogenously in these cells (84). The difference may also reflect the observation that IRFs are principally involved in IFN-β production while NF-κB p65 is more important for the induction of proinflammatory genes upon virus infection (71). In the present study, the expression of a number of NF-κB-dependent genes and ISGs, including IRF7, was found to be depressed by PML-II depletion. IRF7 is essential in the positive-feedback loop of IFN-β expression (85–87). Impaired IRF7 expression would be expected to reinforce the effect of PML-II depletion on IFN-α/β expression and thus amplify the negative effect of this depletion on ISG expression.

Several previous studies have shown that PML proteins generally, or PML-II in particular, play a role in antiviral responses and are targets for viral proteins that are involved in combatting innate immune responses (81). Ad5 E4 Orf3 rearranges PML NB through an interaction with PML-II (47) and is also necessary for the virus to replicate in cells with an established IFN response (34, 35). HSV-1 causes global degradation of PML protein, including PML-II, which is one of two isoforms that are inhibitory to HSV-1 infection (37), via its ICP0 protein. The growth defect of ICP0 mutants *in vivo* is greatly reduced in mice lacking a functional IFN response (32), and the inhibitory effect of IFNs on the replication of ICP0 mutants is largely abolished when they are grown in PML-null cells, in contrast to what is seen in normal cells (88). Furthermore, HSV-2 alters PML RNA splicing to favor PML-V expression over that of PML-II, suggesting a particular significance of PML-II in antiviral responses (89). PML was also shown to limit the replication and speed/extent of pathogenesis of lymphocytic choriomeningitis virus (LCMV) and vesicular stomatitis virus (VSV) in mice, effects suggested to be via enhanced innate immune responses (90). Our findings here are consistent with these previous reports that PML is important for aspects of the antiviral response and provide a mechanistic explanation for this effect.

Numerous proteins that can be physically and/or functionally linked to PML protein have been found (20, 21); in the present study, PML-II was found to associate with transcription factors IRF3, NF-κB, and STAT1 and coactivator CBP. CBP plays an important role in the transcription of a large number of genes, including IFN-β and ISGs (78, 91–93). It provides histone acetyltransferase (HAT) activity, which modifies chromatin and supports recruitment of the general transcriptional machinery, including RNA pol II (94–96). Our finding that the depletion of PML-II causes a significant effect on CBP recruitment to the IFN-β promoter under conditions where its activity should be stimulated therefore contributes to an explanation of its reduced expression. Modulation of CBP function by PML has been suggested previously from studies of hormone signaling (73). We further showed that the ability of PML-II mutants to associate with STAT1 and NF-κB correlated with their ability to potentiate gene expression activated by these factors.

It has been reported that the Ad5 E1A 13S transcriptional activator protein interacts specifically with PML-II to potentiate E1A transcriptional activation (97). In that study, while total PML depletion modestly increased virus yield, restoration of PML-II in a PML-null background caused a substantial further increase. Interpreting these results in light of our findings, we suggest that Ad5 has evolved to make positive use of a PML-II function that intrinsically has antiviral effects when expressed in the context of a full set of PML isoforms. E1A binds CBP and its homologue p300 via its CR1 region and binds various DNA-binding transcription factors via its CR3 region (98, 99). The interaction of E1A with PML-II also required CR3 sequences (97). As these findings are analogous to the effects of PML-II on TF/CBP association with IFN-β/ISG promoters that we observed, we therefore suggest that PML-II also acts in E1A activation by bridging these factors to support their binding to DNA.

The interaction of CBP with PML has been reported to involve sequences from the N-terminal coiled-coil domain that is present in all the principal nuclear PML isoforms (73), indicating that CBP binding should not be restricted to PML-II. However, while we have not tested a full range of isoforms, our data show that PML-II has a functional role in CBP activity that at least one other isoform, PML-V, does not possess. Possibly, the differing PML isoform C-terminal domains affect the conformation or availability of the CBP binding site so that its activity is manifest only in certain isoforms. However, we favor an interpretation in which CBP binding by PML is not sufficient for it to exert a positive effect on transcriptional activity, other sequences that are unique to PML-II also being required. As discussed below, these include sequences that we have shown are necessary for interaction with specific DNA-binding transcription factors.

Different PML isoforms can have distinct functions mediated by their unique C-terminal domains (80). Bioinformatic analysis showed that this part of PML-II was likely to be unstructured but with the propensity to become ordered upon interaction with partner proteins; mutational analysis revealed that interaction with Ad5 E4 Orf3 required one particular molecular recognition element in this region (47). Based on its chemistry and its localization properties, the PML-II unique C-terminal domain was more recently speculated to interact with transcription factors (100). Here we have shown that specific sequences in the unique PML-II C-terminal domain are essential for its interaction with two transcription factors, NF-κB and STAT1, while the N-terminal RBCC domain is dispensable. It was recently reported that the unique C-terminal domain of PML-II can bind to PML-NBs independent of the shared N-terminal region (100), suggesting that C-terminal PML-II might replace full-length PML-II for some functions. In this context, we found that removal of the RBCC domain gave a protein with enhanced activity in the expression of IFN-β and ISGs; the biological significance and mechanism of this effect remains to be determined, but it could reflect a greater functional availability of the PML-II C-terminal domain when less tightly tethered to PML-NB.

In summary, our results show that PML-II positively regulates the expression of genes involved in the IFN response, reflecting a positive effect on the formation of relevant transcription factor complexes and their association with the promoters of these genes. Our data support a model in which activators of the innate immune response cause PML-II to associate with multiple transcriptional complexes and that this interaction facilitates their loading onto, or stable association with, target promoters. The observed role of PML-II in regulating expression of a large number of cytokines and chemokines strongly supports a broad function for PML-II in the innate immune response.
